# SRF and Yap1, partners in cardiac repair

**DOI:** 10.20517/jca.2022.23

**Published:** 2022-08-05

**Authors:** Maha Abdellatif

**Affiliations:** Department of Cell Biology and Molecular Medicine, Rutgers New Jersey Medical School, Newark, NJ 07103, USA.

Therapeutic strategies for the repair of myocardial ischemic damage are an ongoing challenge for both scientists and clinicians. The obstacle is the limited capacity of the terminally differentiated myocytes to proliferate, mainly due to postnatal downregulation of cell cycle proteins and physical hindrance from the perpetually contracting sarcomeres that occupy most of the cells’ volume. Thus far, some of the strategies employed to undertake this challenge include stem cell implantation or injection, inducing myocyte proliferation, or tissue grafting. However, to date, cardiac ischemic damage remains irreparable. Approaches to induce the myocyte to proliferate include suppressing the cyclin-dependent kinase inhibitors (CDKi) by overexpressing a dominant negative FOXO1 or deletion of Meis1, both of which are known to increase CDKi’s^[1]^. Alternatively, overexpression of cyclins-CDKs (CDK1, CDK4, cyclin B1, and cyclin D1) partners efficiently enhanced myocyte proliferation, as previously reported by Mohamed *et al*. ^[2]^. These genes were delivered locally via recombinant adenovirus, which, unfortunately, is unsuitable for gene therapy due to its immunogenicity. Another mechanism involves Yap and TAZ, which activate the transcription of cell cycle proteins, where overexpression of a constitutively active YAP enhances adult myocyte proliferation^[3]^. Uniquely, Xiao *et al.*, in this issue, combined an SRF153(A3) mutant, STEMIN, which lacks the ability to bind the CArG box, with the cell cycle regulator Yap1^[4]^. With this combination, STEMIN induces sarcomere disassembly and dedifferentiation of cardiac myocytes, while YAP increases the expression of the necessary cell cycle proteins, which proved to have a synergestic proliferative effect on the cardiac myocytes. Impressively, intramyocardial injections of the mRNA of both molecules, 5 min after coronary artery occlusion, reduced infarct size and substantially improved ejection fraction. Alone, however, neither molecule was effective.

Gene delivery to the heart and cardiac myocytes also imposes a challenge, as the commonly utilized recombinant adenoviruses or adeno-associated viruses have their limitations. The former is known for its immunogenicity, while the latter is its longevity. When forcing the myocytes to enter the cell cycle, one of the issues that must be addressed is how to terminate the stimulus in order to allow the proliferating myocytes to differentiate. The authors astutely addressed this dilemma by delivering the short-lived synthetically modified mRNA (mmRNA) of the genes, combined with liposomes, intramyocardially. This approach was first reported by Zangi *et al.*, who showed that intramyocardial injection of mmRNA for vascular endothelial growth factor A (VEGF-A) improved cardiac function in mice with myocardial infarction^[5]^. Notably, injecting the mmRNA proved superior to injecting the DNA of the gene. Since then, this technology has gained traction, as one of its most recognized uses has been the development of the COVID19 vaccines^[6]^. To sum up, the combination of STEMIN, YAP5SA, and intramyocardial mmRNA delivery proved to be an effective approach for inducing myocyte proliferation and myocardial repair of the ischemic heart.
